# Features of the Defect Structure of the Compositionally Homogeneous Crystal LiNbO_3_:Er^3+^(3.1 wt%) and the Gradient Crystal LiNbO_3_:Er^3+^ and Their Manifestation in the IR Transmission Spectra in the Region of Stretching Vibrations of Hydrogen Atoms of OH^−^-Groups

**DOI:** 10.3390/ma18030579

**Published:** 2025-01-27

**Authors:** Nikolay Sidorov, Alexander Pyatyshev, Elena Stroganova, Valeriy Galutskiy, Andrey Bushunov, Mikhail Tarabrin

**Affiliations:** 1 Tananaev Institute of Chemistry—Subdivision of the Federal Research Centre, Kola Science Centre of the Russian Academy of Sciences (ICT RAS), 184209 Apatity, Russia; n.sidorov@ksc.ru; 2 P. N. Lebedev Physical Institute of the Russian Academy of Sciences, 119991 Moscow, Russia; 3 Department of Optoelectronics, Kuban State University, Stavropolskaya 149, 350040 Krasnodar, Russia; stroganova@phys.kubsu.ru (E.S.); galutskiy17v@mail.ru (V.G.); 4 Infrared Laser Systems Laboratory, Bauman Moscow State Technical University, 2nd Baumanskaya Str., 5, b. 1, 105005 Moscow, Russia; zendel@yandex.ru (A.B.); tarabrinmike@yandex.ru (M.T.)

**Keywords:** lithium niobate, Czochralski method, erbium doping, composition gradient, IR transmission spectrum, OH^−^-group, Klauer’s method, point defect, nonlinear material

## Abstract

Based on the analysis of the IR transmission spectra in the region of stretching vibrations of hydrogen atoms of OH^−^-groups, it was established that the oxygen-octahedral MeO_6_ clusters (Me-Li^+^, Nb^5+^, vacant octahedron V, impurity ion) of the structure of the compositionally homogeneous crystal LiNbO_3_:Er^3+^(3.1 wt%) and the gradient crystal LiNbO_3_:Er^3+^(congruent composition by the main components, Er gradient of 0.55 at%/cm) have a shape close to the regular one. In this case, the value of R = [Li]/[Nb] ≈ 1, and in the structure of both crystals, there are practically no point defects in Nb_Li_ responsible for the photorefraction effect. By using the IR transmission spectra and Klauer’s method, it was found that the volume concentration of OH^−^-groups in the gradient crystal LiNbO_3_:Er^3+^ is almost an order of magnitude lower than in the compositionally homogeneous LiNbO_3_:Er^3+^(3.1 wt%) crystal. This fact explains the lower hydrogen conductivity of the gradient crystal LiNbO_3_:Er^3+^ and the lower photorefraction effect compared to the compositionally homogeneous LiNbO_3_:Er^3+^(3.1 wt%) crystal. The results obtained are important for the development of materials for active nonlinear laser media and for the conversion of laser radiation.

## 1. Introduction

Nonlinear optical lithium niobate single-crystal (LiNbO_3_) has a number of unique properties, which ensures its wide application as functional elements of modern optoelectronics, telecommunication devices and integrated and laser optics [1,2,3,4,5]. At the same time, for the production of functional elements in industry, compositionally homogeneous LiNbO_3_ single-crystals of congruent composition (R = [Li]/[Nb] = 0.946), both nominally pure and doped, are mainly used.

In recent years, a new direction has been intensively developing in materials science related to the creation of structures with physical characteristics that change in volume: magnetic [6], ferroelectric [7] and piezoelectric [8]. Along with the predicted change in the ferroelectric characteristics of such systems compared to homogeneous samples (e.g., a change in the phase transition), a number of unique effects were discovered in them. For example, when placing a sample with a composition gradient in the Sawyer–Tower scheme, a significant shift of the hysteresis loop along the polarization axis is observed. Based on this effect, a device was developed that allows for charge amplification up to 150 times under external energy impact. In this case, the type of energy impact can be different—thermal, deformation, or electrical [9,10]. In a number of such systems, a “giant” pyroelectric effect is observed, in which the pyroelectric response is 10–100 times higher than the signal of homogeneous ferroelectrics of the corresponding composition [11,12]. The axial doping gradient of some laser materials allows for a significant reduction in thermal loads and an increase in the output power of laser radiation [13,14,15]. For LuAG:Ce-LuAG scintillator crystals, the composition gradient allowed us to increase the integral luminescence intensity [16]. The composition gradient allows us to provide optimal conditions for the growth of semiconductor solid solutions [17] and control a number of dielectric and magnetic characteristics of ceramics and crystals [18,19,20,21]. Some results in this direction are summarized in the reviews [22,23]. In this case, the directed changes in the physical characteristics of the material are determined by the spatial change in the composition of the material. Such materials are called “gradient materials”. Materials with a gradient refractive index are used, in particular, in the creation of film waveguides [24] and sensors [25,26].

To date, studies of the optical properties of gradient LiNbO_3_:Er^3+^ crystals have been primarily focused on the analysis of luminescence. In [27], for a double-doped gradient crystal LiNbO_3_:Er^3+^:Yb^3+^, a multidirectional change in the intensity of green (525–585 nm) and IR luminescence (1400–1700 nm) was found depending on the concentration of erbium ions. This difference was explained [28] by various lifetimes and mechanisms of population of the energy levels of the doping ions. It was found that heating of the double-doped gradient crystal LiNbO_3_:Er^3+^:Yb^3+^ in the range of 300–473 K leads to a change in the refractive indices [29] and, unusually, to an increase in the luminescence intensity by 15–20% in the range of 1480–1680 nm. At the same time, for the single-doped gradient crystal LiNbO_3_:Er^3+^, a decrease in the luminescence intensity by 30% is observed with increasing temperature [30]. Based on the double-doped gradient crystal LiNbO_3_:Er^3+^:Yb^3+^, a design of proton-exchange waveguides was proposed [31]. In the article [31], a change in the absorption spectrum was discovered for a double-doped crystal LiNbO_3_:Er^3+^:Yb^3+^ with a rare-earth ion concentration gradient, correlating with a change in the composition concentration along the crystal growth axis. However, the configuration of the oxygen-octahedral clusters MeO_6_ and the state of defects of the gradient crystals were not analyzed. Meanwhile, the degree of distortion of the MeO_6_ clusters and the features of the distribution of the main (Li^+^ and Nb^5+^) and doping cations over the O_6_ octahedra determine the position of the Er^3+^ ions and polarons ${\mathrm{Nb}}_{\mathrm{Li}}^{4+}$ in the LiNbO_3_:Er^3+^ crystal and, accordingly, the intensity of luminescence and laser generation. In particular, the Er^3+^ ion in the LiNbO_3_ crystal matrix is a very effective emitter in the visible and IR spectral regions [32,33,34]. For this reason, LiNbO_3_:Er^3+^ crystals successfully combine the excellent nonlinear optical properties of the nominally pure LiNbO_3_ matrix and the laser characteristics of the Er^3+^ ion. Radiation with wavelengths of 980 and 1480 nm is effectively absorbed by the erbium ion, causing excess heat generation inside the crystal. Excess heat generation affects the temperature conditions that ensure radiation focusing in the crystal. Thus, at a significant value of pump radiation, heat will be generated in LiNbO_3_:Er^3+^, violating the parameters of the pump radiation propagation medium. Uneven distribution of the thermal field along the length of the element leads to the formation of “thermal lenses”, which entails the effect of changing the refractive index and defocusing of the laser radiation, and “thermal shock”—the destruction of the active element structure and, ultimately, the crystal itself. The gradient distribution of Er^3+^ ions in the crystal suggests a change in the ion concentration along the length of the active element and uniform heat generation of excess energy throughout the crystal. Thus, the composition gradient in the LiNbO_3_:Er^3+^ crystal allows for compensation of temperature distortions and creates optimal conditions for laser generation and for the conversion of laser radiation [29,30]. In addition, compositionally homogeneous and gradient LiNbO_3_:Er^3+^ crystals are characterized by a low photorefraction effect, comparable to that of the congruent crystal LiNbO_3cong_ [32], which is important for the development of laser materials and materials for converting laser radiation.

When growing gradient LiNbO_3_ crystals (both nominally pure and doped), the composition gradient is realized under highly nonequilibrium conditions in accordance with the incongruent melting of the crystal as a non-stoichiometric phase of variable composition and the features of the phase diagram for a specific composition [27]. It would seem that gradient LiNbO_3_ crystals, obtained under more nonequilibrium crystallization conditions (compared to compositionally homogeneous crystals), should be characterized by an increased content of various types of point and spatial defects, significantly reducing the quality of the optical material. However, there is reason to believe that this is not always the case. The features of certain chemically active complexes in the melt [35,36,37], which determine the features of localization in the oxygen-octahedral MeO_6_ clusters (Me-Li^+^, Nb^5+^, vacant octahedron V, impurity ion) of the LiNbO_3_ crystal of the main (Li^+^, Nb^5+^) and doping ions [32] (as well as hydrogen atoms bound to oxygen atoms by hydrogen bonds [38,39,40]), under certain conditions can lead to a more perfect crystal structure of both doped compositionally inhomogeneous crystals and gradient LiNbO_3_ crystals. Crystals with a more perfect structure and a lower photorefraction effect are more preferable for the development of functional materials for active nonlinear laser media and for the conversion of laser radiation.

The main structural defects that have a significant effect on the physical characteristics of the LiNbO_3_ crystal are point defects in the form of basic cations (Li^+^, Nb^5+^) located in the oxygen octahedra O_6_, not in their positions and cations of impurity metals [41], as well as microinclusions of impurity phases of other lithium niobates and cluster defects. An important type of defect formed in the LiNbO_3_ crystal during its growth by the Czochralski method in an air atmosphere is complex defects caused by the presence of hydroxyl OH^−^-groups in the structure [38,39,40,41,42]. When growing LiNbO_3_ crystals in an air atmosphere, hydrogen contained in water vapor interacts with oxygen atoms of oxygen-octahedral MeO_6_ clusters of oxygen octahedra O_6_ of the LiNbO_3_ crystal structure, forming hydrogen bonds, which leads to the formation of crystal lattice defects in the form of hydroxyl OH^−^-groups. It should be noted that in the ideal structure of a LiNbO_3_ crystal of strictly stoichiometric composition (R = 1), in which there are no Nb_Li_ point defects, the placement of hydrogen atoms is impossible [38].

The arrangement of hydrogen atoms in the structure of real LiNbO_3_ crystals changes depending on the value of the R, concentration and type of dopant [38,39,40,41]. In this case, the configuration of oxygen-octahedral clusters of the MeO_6_ structure, responsible for spontaneous polarization, nonlinear optical and ferroelectric properties of the crystal, is disrupted. The presence of hydrogen in the structure of the LiNbO_3_ crystal leads to the formation of complex defects: V_Li_-OH, Nb_Li_-OH, Me-OH, Me-OH-Me, etc., and to a change in its nonlinear optical and ferroelectric characteristics, affecting such practically significant properties of the LiNbO_3_ crystal as photoluminescence, photorefractive thermal fixation, dark conductivity and photorefractive sensitivity [41].

The frequency of the stretching vibration of the hydrogen atom in the hydroxyl OH^−^-group of the heterodesmic LiNbO_3_ crystal, due to the smallness of the hydrogen bond compared to the covalent (Nb-O) and electrostatic (Li-O) bonds in the oxygen-octahedral MeO_6_ clusters (Me-Nb, Li, vacancy V, impurity ion), is extremely sensitive to the slightest changes in the crystal field, caused, among other things, by the composition gradient. Therefore, the methods of vibrational spectroscopy (IR absorption spectroscopy and Raman spectroscopy) in the region of stretching vibrations of hydrogen atoms of OH^−^-groups can be very effective for obtaining information on the subtle features of the defect structure of the LiNbO_3_ crystal. In this case, the IR absorption spectra are much more sensitive to the presence of OH^−^-groups than the Raman spectra, due to the strong absorption of infrared radiation. This method can be used to study the diffusion processes of hydrogen and deuterium [43,44], to numerically evaluate the efficiency of increasing the volume concentration of hydrogen [45], analyze the depth of ion penetration during diffusion alloying with metals [46], and to analyze concentration thresholds [47].

In this work, comparative studies of the state of defects of the gradient crystal LiNbO_3_:Er^3+^(congruent composition by the main components, Er gradient of 0.55 at%/cm) (cLiNbO_3_:Er^3+^(0.55 at%/cm)) are performed for the first time using IR transmission spectra in the region of stretching vibrations of hydrogen atoms of hydroxyl OH^−^-groups. Compositionally homogeneous LiNbO_3_:Er^3+^(3.1 wt%) single-crystal was used as a comparison sample. It should be noted that the concentration of 3.1 wt% of Er^3+^ ions in the compositionally homogeneous LiNbO_3_ crystal was not chosen by chance. It is known that the LiNbO_3_:Er^3+^ crystal is characterized by concentration thresholds at which the mechanism of Er^3+^ impurity entry into the structure and the physical characteristics of the crystal change abruptly [32,33]. In [32], it was established that for “post-threshold” LiNbO_3_:Er^3+^ crystals ([Er] > 2.5 wt%), a significant decrease in the Er distribution coefficient is observed, and it approaches unity. Consequently, LiNbO_3_:Er^3+^ crystals ([Er] > 2.5 wt%), including the LiNbO_3_:Er^3+^(3.1 wt%) crystal, will have higher compositional and optical homogeneity than crystals with a lower erbium content, since for the latter, the distribution coefficient is significantly higher than unity.

## 2. Materials and Methods

**Figure 1:**
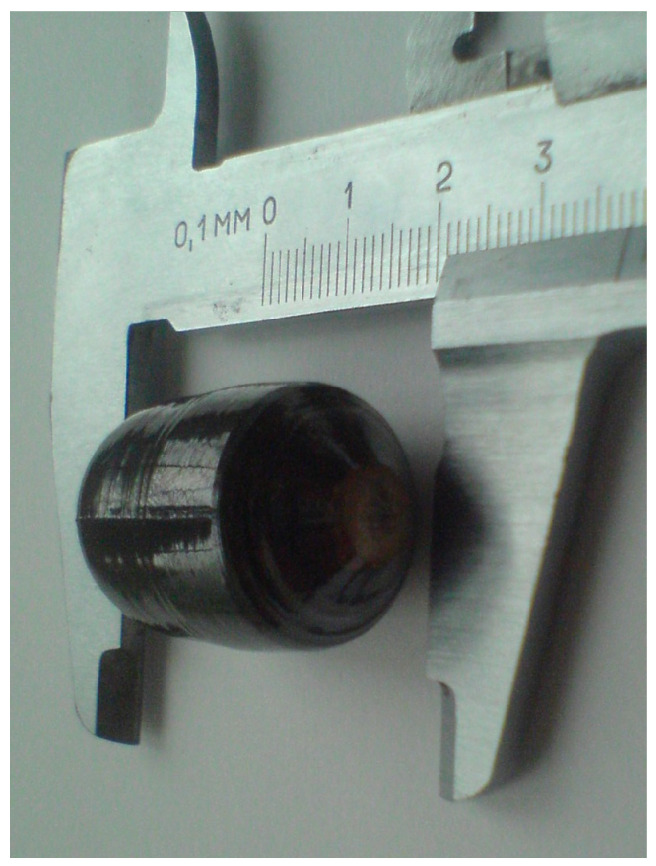
Photo of the grown gradient crystal cLiNbO_3_:Er^3+^(0.55 at%/cm).

To grow a gradient crystal cLiNbO_3_:Er^3+^(0.55 at%/cm), the Czochralski method with liquid feeding (double crucible method) was used [27]. A system of platinum crucibles inserted into each other was used: an outer (main crucible) with a diameter of 8 cm and an inner (crucible reactor) with a diameter of 5.2 cm. The system of crucibles moved relative to each other. Setting the speed of movement of the crucible reactor relative to the main crucible is necessary to maintain the concentration gradient of Er^3+^ ions in the LiNbO_3_ crystal specified before growth. The composition of LiNbO_3_, congruent in the main components, was preserved during the relative movement of the crucibles with the melt. Before pulling the crystal and fusing the melt, the type of distribution profile of the laser impurity Er^3+^ in the crystal was specified as a function of the coordinate of the crystal along the length. The crystal pulling rate was set before the start of growth, and its value was determined by the relaxation of the melt composition at the crystallization front. The relaxation of the melt composition was determined by the change in the Er^3+^ ion concentration at the crystallization front over time due to the melt being fed into the crucible reactor with melt from the main crucible with a different Er^3+^ concentration. Figure 1 shows a photograph of the grown gradient crystal cLiNbO_3_:Er^3+^(0.55 at%/cm).

Niobium oxide (purity 99.97%), erbium oxide (purity 99.99%) and lithium carbonate (purity 99.3%) (all reagents are produced by JSC «Vekton», Saint Petersburg, Russia) taken in the required mass ratios to obtain a compound with the chemical formula LiNbO_3_ of congruent composition were used to prepare the charge [27]. The charge was tableted at a pressure of 60–120 atmospheres in plexiglass press molds. The resulting tablets were placed in a muffle furnace for solid-phase synthesis for 24 h at a temperature of 900 °C. To grow LiNbO_3_ crystals with the Er^3+^ concentration profile, the charge was melted in the form of tablets in a crucible system. The grown single-crystal was annealed in a muffle furnace at a temperature of 1200 °C for 5 h. The technology for obtaining gradient crystals cLiNbO_3_:Er^3+^(0.55 at%/cm) is presented in more detail in [27]. A plate of about 3 mm thickness was cut from the grown crystal. This plate was cut along the entire length of the crystal. The plate was cut along the Z axis; the X axis was directed perpendicular to the plate. Then, this plate was cut into 9 areas evenly spaced from each other. The size of the areas is justified by the dimensions of the aperture for measuring the Raman and IR transmission spectra. The number of studied areas of the cLiNbO_3_:Er^3+^(0.55 at%/cm) reflects the number of measurements required to track the dynamics of the transformation of the properties of Er^3+^ impurity centers with a change in the Er^3+^ concentration in the crystal. With an increase in the step (size) of the crystal samples, as a result of optical measurements, an integral characteristic along the growth direction (Z axis) was realized, while in the X or Y measurement direction, the type and quantitative relationships between the characteristics would change inside the crystalline sample. Therefore, the thickness of the plates in the X axis direction (due to the absence of a concentration gradient in this direction) was related to the step (thickness) of the crystal plate along the Z axis (where the concentration gradient was present). Area 1 corresponded to the initial point of the crystalline boule; the concentration of Er^3+^ ions in it was 3.0 at%. With an increase in the area number, the concentration of Er^3+^ ions decreases with a given gradient of 0.55 at%/cm.

The comparison sample, a compositionally homogeneous LiNbO_3_:Er^3+^(3.1 wt%) single-crystal, was grown by the Czochralski method in an air atmosphere in a platinum crucible under conditions of a comparatively small (2–4 °C/cm) axial temperature gradients in the direction of the polar Z axis at constant rotation (16 rpm) and movement (0.8 mm/h) speeds [32]. During crystal growth, the crystallization front was flat. A high-purity, chemically homogeneous, single-phase, granulated lithium niobate batch of congruent composition with high bulk density, developed at the Tananaev Institute of Chemistry—Subdivision of the Federal Research Centre, Kola Science Centre of the Russian Academy of Sciences (Technical Specification No. 0.027.039), was used. The alloying element Er^3+^ was introduced into the batch immediately before deposition in the form of Er_2_O_3_ (purity 99.99%, JSC «Solikamsc Magnesium Plant», Solikamsk, Russia). The melt for homogenization of impurity in it before the beginning of crystal growth was maintained for 6 h under conditions of overheating by 150 °C. The grown single-crystal was annealed at a temperature of 1200 °C for 5 h. The heating rates of the batch and cooling of the crystal were the same—50 °C/h. The concentration of erbium ions in the LiNbO_3_:Er^3+^(3.1 wt%) crystal was determined by analyzing plates cut in the upper (conical) and lower (end) parts of the single-crystal boule, using the emission spectrometry method using a spectrometer ICPE-9000 (Shimadzu, Kyoto, Japan, 2011).

The LiNbO_3_:Er^3+^(3.1 wt%) single-crystal was monodomainized by high-temperature electrodiffusion annealing with the application of constant electric voltage during the cooling of the samples at a rate of 20 °C/h in the temperature range of 1240–890 °C. The technology for obtaining a compositionally homogeneous LiNbO_3_:Er^3+^(3.1 wt%) single-crystal, used in this work as a comparison sample, is presented in more detail in [32].

The IR transmission spectra in the region of stretching vibrations of the OH^−^-groups were recorded using a Bruker VERTEX 70x spectrometer (Bruker, Karlsruhe, Germany) with a spectral resolution of 0.4 cm^−1^. The measurements were carried out taking into account the reflection of all elements and the absorption of the optical path. The photometric accuracy of the spectrometer used is better than 0.1%. The measurements were carried out in a vacuum at a pressure of 1.78 hPa (in order to eliminate the absorption lines of the atmosphere) and room temperature.

Raman spectra were recorded using a BWS465-785H i-Raman Plus spectrometer (B&W Tek, Plainsboro Township, NJ, USA) using a known technique [48,49,50]. All spectra were recorded at 293 K.

## 3. Results

**Figure 2:**
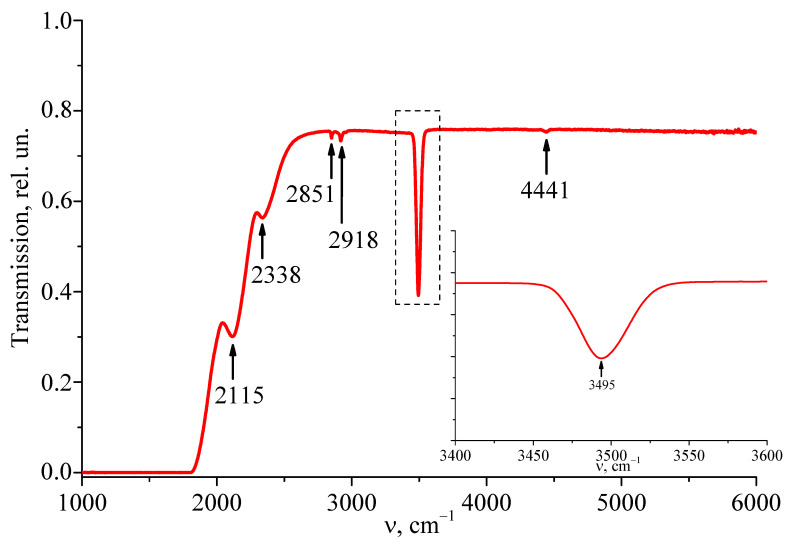
IR transmission spectrum of a compositionally homogeneous LiNbO_3_:Er^3+^(3.1 wt%) crystal in the region of 1000–6000 cm^−1^. The inset shows an enlarged view of the IR transmission spectrum in the region of stretching vibrations of OH^−^-groups.

**Figure 3:**
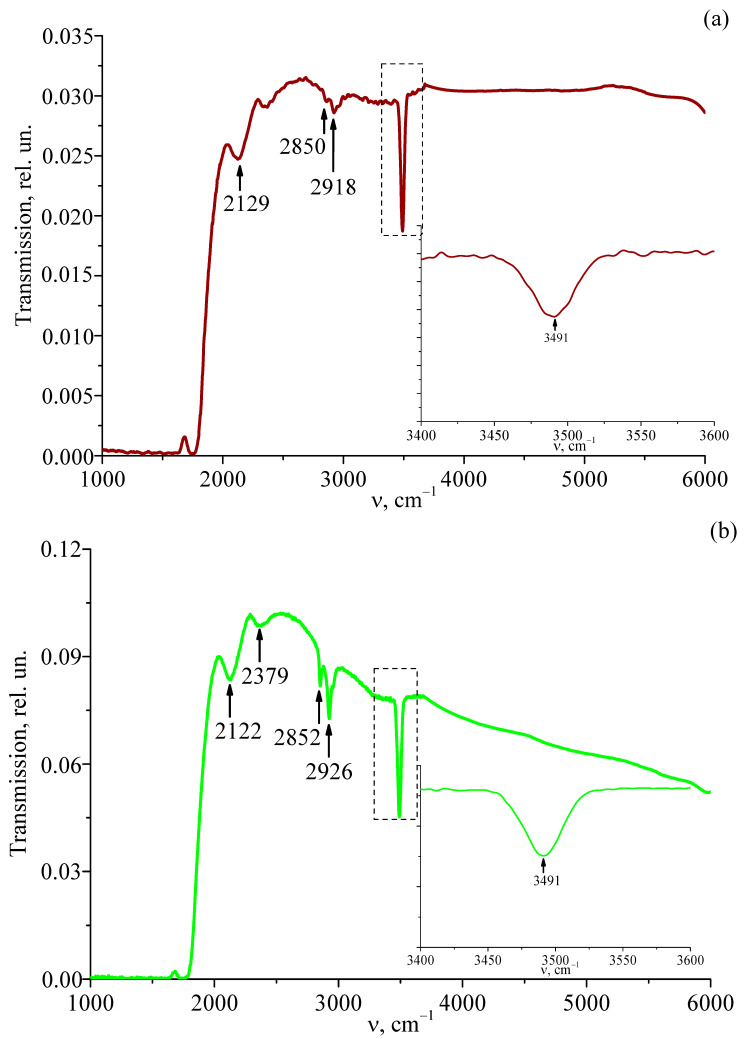

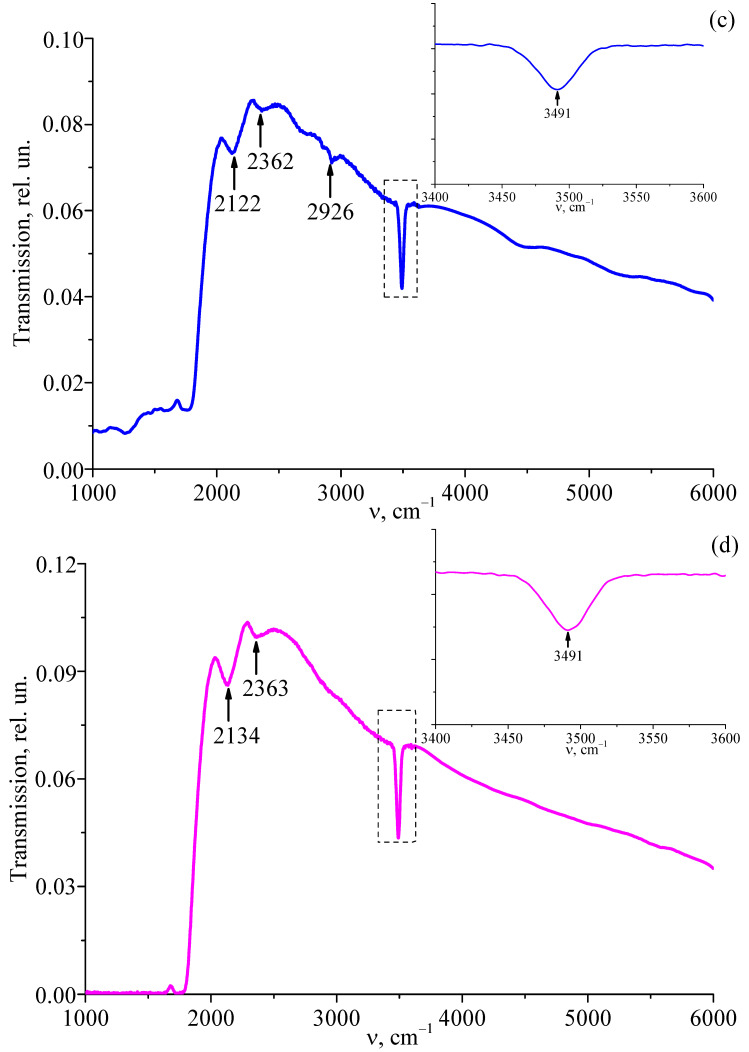

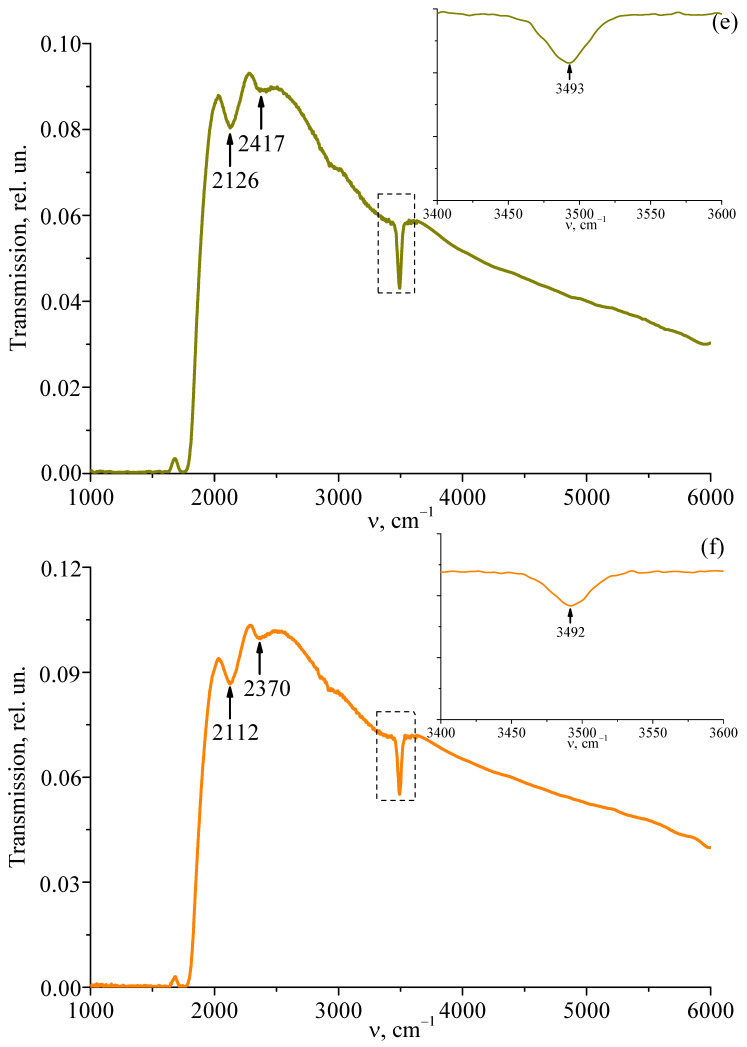

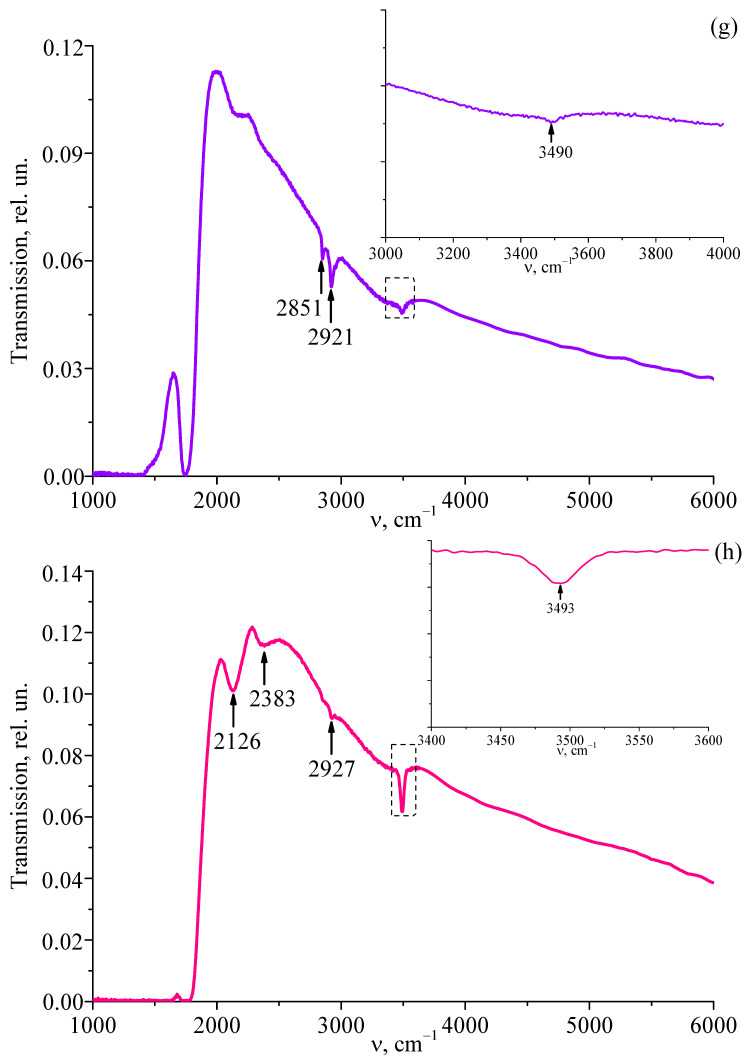

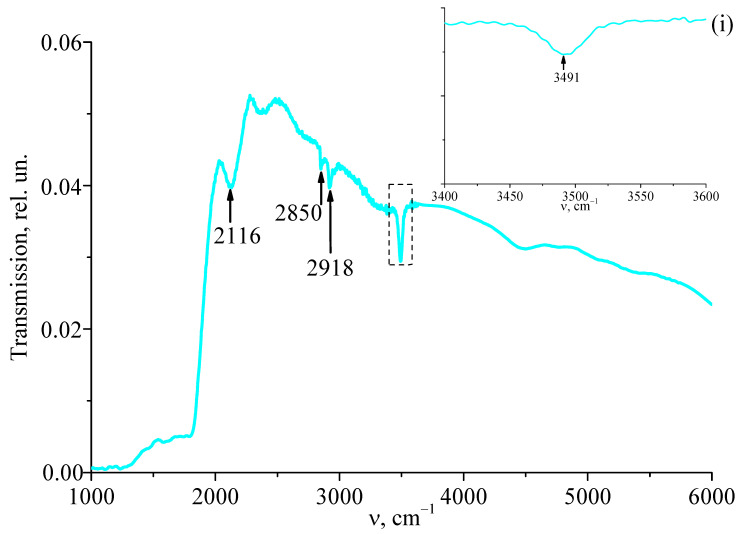
IR transmission spectra from different areas of the gradient crystal cLiNbO_3_:Er^3+^(0.55 at%/cm): area 1 (**a**), area 2 (**b**), area 3 (**c**), area 4 (**d**), area 5 (**e**), area 6 (**f**), area 7 (**g**), area 8 (**h**) and area 9 (**i**) in the region of 1000–6000 cm^−1^. The insets show an enlarged view of the IR transmission spectrum in the region of stretching vibrations of OH^−^-groups.

Figure 2 and Figure 3 illustrate the IR transmission spectra in the range of 1000–6000 cm^−1^ of the compositionally homogeneous LiNbO_3_:Er^3+^(3.1 wt%) single-crystal and the gradient crystal cLiNbO_3_:Er^3+^(0.55 at%/cm). IR transmission spectra were recorded along the ferroelectric axis of the crystal.

As can be seen from Figure 2, several lines are clearly visible in the IR transmission spectrum of the LiNbO_3_:Er^3+^(3.1 wt%) crystal. The line with a frequency of 3495 cm^−1^ corresponds to the stretching vibrations of the hydrogen atom of the hydroxyl OH^−^-group in the cluster defect site Er^3+^ (${\mathrm{Er}}_{\mathrm{Li}}^{2+}$-OH-${\mathrm{Er}}_{\mathrm{Nb}}^{2-}$), which consists of one Er_Li_ point defect (Er^3+^ ion in the position of the Li^+^ ion) and one Er_Nb_ point defect (Er^3+^ ion in the position of the Nb^5+^ ion) of the ideal LiNbO_3_ structure of stoichiometric composition [51]. For double-doped crystals, this absorption band shifts by ≈10 cm^−1^ towards lower frequencies [51,52]. The absorption lines with frequencies of 2851 and 2918 cm^−1^ correspond to the symmetric and antisymmetric stretching vibrations of the hydrogen atoms of the C-H bond of the CH_2_ group. Compounds with CH_2_ groups can be present in microquantities on the crystal surface due to wiping the crystal surface with ethyl alcohol after polishing. The absorption lines with frequencies of 2115 and 2338 cm^−1^ correspond to the absorption bands of the CO and CO_2_ groups. Their origin can be associated with the presence of lithium carbonate, from which a batch is prepared for growing lithium niobate crystals.

Figure 3 presents the IR transmission spectra recorded from different areas of the gradient crystal cLiNbO_3_:Er^3+^(0.55 at%/cm). IR transmission spectra were recorded along the ferroelectric axis of the crystal. Comparison of Figure 2 and Figure 3 shows that the IR transmission spectra of the compositionally homogeneous LiNbO_3_:Er^3+^(3.1 wt%) crystal and the gradient crystal cLiNbO_3_:Er^3+^(0.55 at%/cm) differ significantly. The gradient crystal has a much higher absorption in the frequency range of 2000–4000 cm^−1^. At the same time, a characteristic feature of the compositionally homogeneous LiNbO_3_:Er^3+^(3.1 wt%) crystal and the gradient crystal cLiNbO_3_:Er^3+^(0.55 at%/cm) studied by us is that the line corresponding to the stretching vibrations of hydrogen ions in the OH^−^-group is not split into separate components (inserts in Figure 2 and Figure 3), as is observed for many compositionally homogeneous non-stoichiometric LiNbO_3_ crystals, both nominally pure and doped with a wide range of different metals [38,39,40,41].

The IR transmission spectrum also contains a number of lines characteristic of the batch components or technological processes for growing gradient crystals cLiNbO_3_:Er^3+^(0.55 at%/cm). The absorption lines with frequencies of 2112–2134 and 2362–2363 cm^−1^ correspond to the absorption bands of the CO and CO_2_ groups. Thus, the absorption bands of 2850–2852 and 2916–2927 cm^−1^ correspond to symmetric and antisymmetric stretching vibrations of the hydrogen atoms of the C-H bond of the CH_2_ group.

The significant absorption of the gradient crystal cLiNbO_3_:Er^3+^(0.55 at%/cm) may be due to the fact that its structure is closer to polycrystalline than to monocrystalline. A less intense absorption band is observed in the region of the stretching vibrations of OH^−^-groups. This may be due to the design feature of the thermal unit in which the gradient crystal is grown. Despite the wide range studied (1000–6000 cm^−1^), differences are observed mainly in the region of stretching vibrations of OH^−^-groups of the studied crystals. This fact indicates a special role of hydroxyl groups in the formation of structural features and properties of LiNbO_3_:Er^3+^ crystals of different genesis.

In the IR transmission spectrum and in the Raman spectrum of non-stoichiometric, non-gradient crystals of different compositions, the lines corresponding to the stretching vibrations of the hydrogen atoms of the OH^−^-groups are observed in the frequency range of 3450–3550 cm^−1^ [38,39,40]. In this case, the number of observed lines (provided that the oxygen-octahedral clusters of MeO_6_ are nonequivalent to each other) should not exceed six (according to the number of OH^−^-groups in the MeO_6_ cluster). The lengths of the O-H stretching bonds and, accordingly, their quasi-elastic constants in such nonequivalent MeO_6_ clusters will differ. Therefore, the frequencies of the stretching vibrations of the hydrogen atoms of the OH^−^-groups will also differ. The more perfect (symmetrical) the MeO_6_ cluster, the less the frequencies of the stretching vibrations of the hydrogen atoms of the OH^−^-groups will differ from each other [38]. In the case of a highly perfect stoichiometric crystal, there is only one position for the hydrogen atom, and its IR transmission spectrum exhibits only one line with a frequency of 3466 cm^−1^ [38]. In the structure of most non-stoichiometric LiNbO_3_ crystals, there are more than two positions for hydrogen atoms, and their IR absorption spectrum exhibits more than two lines in the frequency range of 3450–3550 cm^−1^. In particular, in the IR transmission spectrum of the congruent crystal LiNbO_3cong_, according to numerous studies, three lines with frequencies of 3470, 3483 and 3486 cm^−1^ are observed [38,39,40].

The formation of hydrogen bonds in the LiNbO_3_ crystal leads to a noticeable change in the wave functions of the outer electron orbitals of oxygen ions and the parameters of its electron polarizability [39,40,41] and, accordingly, to a noticeable distortion of the entire oxygen-octahedral MeO_6_ cluster. The absence of line components in the IR transmission spectrum corresponding to the stretching vibrations of hydrogen atoms in the OH^−^-groups of various oxygen-octahedral MeO_6_ clusters of the crystal structure indicates that the MeO_6_ clusters in the structure of the LiNbO_3_:Er^3+^(3.1 wt%) crystal and the gradient crystal cLiNbO_3_:Er^3+^(0.55 at%/cm), as well as the MeO_6_ clusters of the highly perfect crystal of stoichiometric composition [36], have a shape close to regular, that is, they are practically undistorted. The results we obtained are a good confirmation of the results of works [32], in which the method of X-ray structural analysis showed that at erbium concentrations in compositionally homogeneous crystals of LiNbO_3_:Er^3+^ of more than 2.66 wt%, a reduction in the difference between the values of long and short distances in the niobium octahedron (NbO_6_) is observed. This fact also indicates that the shape of the oxygen-octahedral MeO_6_ clusters in the crystal structure tends to the correct shape, characteristic of a highly perfect crystal of stoichiometric composition.

**Figure 4:**
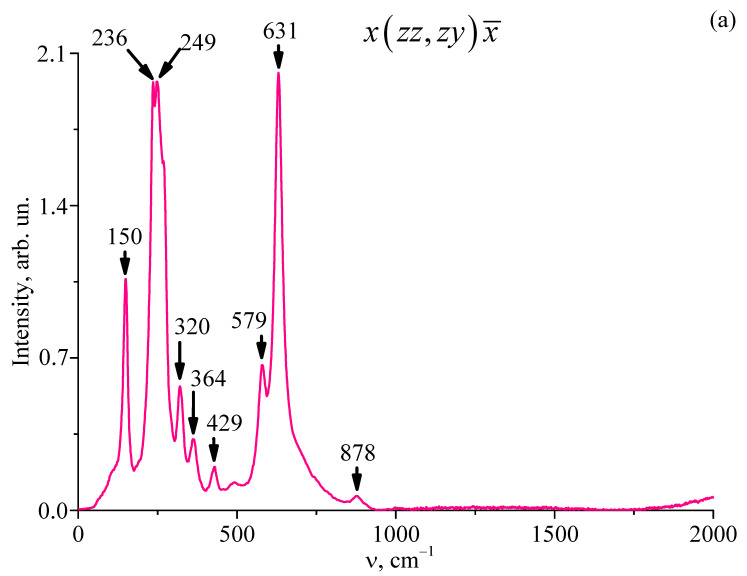

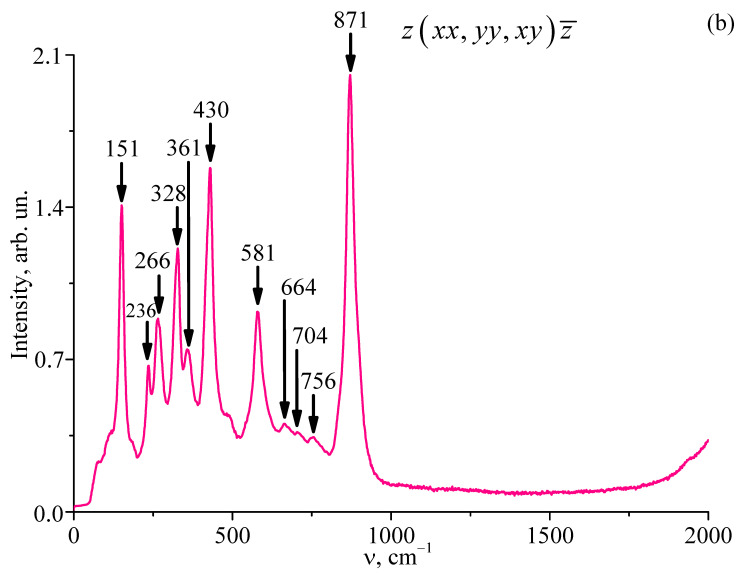
Raman spectra of the LiNbO_3_:Er^3+^(3.1 wt%) crystal in two different backscattering geometries upon excitation by radiation with λ_0_ = 785 nm. (**a**) $x(\mathrm{zz};\mathrm{zy})\bar{x}$ geometry, (**b**) $z(\mathrm{xx};\mathrm{yy};\mathrm{xy})\bar{z}$ geometry.

**Figure 5:**
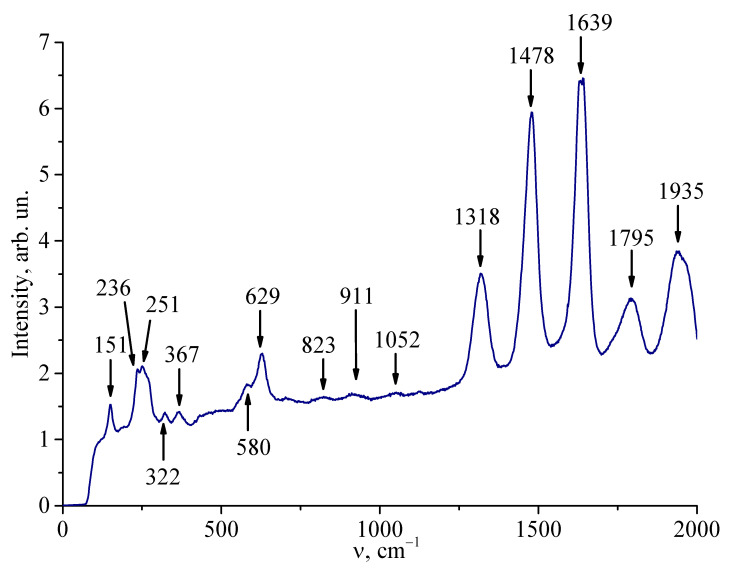
Raman spectrum of the gradient crystal cLiNbO_3_:Er^3+^(0.55 at%/cm) in a wide frequency range, including the range of fundamental vibrations of the crystal lattice (first-order spectrum, 100–1000 cm^−1^) and the second-order spectrum (range 1000–2000 cm^−1^) upon excitation by radiation with λ_0_ = 785 nm.

Thus, in the compositionally homogeneous crystal LiNbO_3_:Er^3+^(3.1 wt%) and in the gradient crystal cLiNbO_3_:Er^3+^(0.55 at%/cm) studied by us, the value of R ≈ 1, i.e., it is close to that for a crystal of stoichiometric composition. The correctness of this conclusion is also confirmed by the absence of a second-order IR transmission spectrum (Figure 2) and a second-order Raman spectrum (Figure 4) in the studied crystal LiNbO_3_:Er^3+^(3.1 wt%) in the frequency range of 1000–2000 cm^−1^. It is widely known that for a highly ordered stoichiometric crystal LiNbO_3stoich_, there is no second-order Raman spectrum in the frequency range of 1000–2000 cm^−1^. But the second-order spectrum is reliably observed for non-stoichiometric LiNbO_3_ crystals, both nominally pure and doped [44,45,46,47]. What is unusual is that in the gradient crystal cLiNbO_3_:Er^3+^(0.55 at%/cm) in the frequency range of 1000–2000 cm^−1^, the second-order Raman spectrum is reliably observed (Figure 5), but the second-order IR transmission spectrum is absent (Figure 3).

If the value of R ≈ 1 in the LiNbO_3_ crystal (near-stoichiometric composition), then the crystal structure should be practically free of Nb_Li_ point defects responsible for the photorefraction effect, and the erbium ions will occupy not one, but two positions in the cation sublattice. Moreover, one of the positions is the main one (Er_Li_) and is populated significantly more than the other position (Er_V_). Indeed, according to the data of X-ray structural analysis, in LiNbO_3_:Er^3+^ crystals with an erbium concentration of more than 2.66 wt%, studied in [32], the proportion of Nb_Li_ point defects is small. Moreover, Nb_Li_ defects at erbium concentrations of more than 2.66 wt% appear due to the displacement of niobium ions by erbium ions from empty octahedra; that is, there is also a decrease in the concentration of Er_V_ point defects. From the obtained results, it becomes clear why the LiNbO_3_:Er^3+^(3.1 wt%) crystal has a low photorefraction effect: it has a low concentration of Nb_Li_ defects responsible for the photorefraction effect [32].

The contribution to the photorefraction effect in LiNbO_3_:Er^3+^(3.1 wt%) crystal and gradient crystal cLiNbO_3_:Er^3+^(0.55 at%/cm) can also be made by OH^−^-groups, since their presence in the crystal increases the electrical conductivity of the crystal, and, accordingly, the photorefraction effect will also increase [36]. Based on the IR transmission spectra, the volume concentration of OH^−^-groups in the studied crystals can be calculated using Klauer’s method [53,54]. The calculation results obtained, taking into account the thickness of the studied gradient samples, are presented in Table 1.

It is evident from Table 1 that the concentration of OH^−^-groups in the gradient crystal cLiNbO_3_:Er^3+^(0.55 at%/cm) does not exceed 7.0 × 10^16^ cm^−3^. At the same time, the concentration of OH^−^-groups in the compositionally homogeneous crystal LiNbO_3_:Er^3+^(3.1 wt%) is 6.7 × 10^17^ cm^−3^, which is almost an order of magnitude greater than in the gradient crystal cLiNbO_3_:Er^3+^(0.55 at%/cm). The concentration of OH^−^-groups in the congruent LiNbO_3cong_ and stoichiometric LiNbO_3stoich_ crystals are, respectively, 3.26 × 10^17^ and 1.58 × 10^17^ cm^−3^. This fact indicates a lower conductivity of the gradient crystal cLiNbO_3_:Er^3+^(0.55 at%/cm) and, accordingly, a lower photorefraction effect, compared to compositionally homogeneous crystals of LiNbO_3_:Er^3+^(3.1 wt%), LiNbO_3cong_ and LiNbO_3stoich_. It should be noted that in the visible region, direct measurements of the photorefraction effect in the gradient crystal cLiNbO_3_:Er^3+^(0.55 at%/cm) are significantly complicated due to strong absorption by the crystal, which has a dark color (Figure 1).

The obtained results are of interest for many reasons. Since the presence of OH^−^-groups play an important role in the formation of the secondary structure and physical characteristics of the crystal, determination of its concentration is necessary to determine the defect structure. In this case, it is much easier to do using IR spectroscopy due to its high sensitivity than using Raman spectroscopy. Hydrogen has its own diffusion coefficient in a single-crystal. Spatial variations in its concentration are possible. In the case of the gradient crystal studied in our work, analysis of the spatial distribution of the OH^−^-group concentration provides information on the change in the ${\mathrm{Er}}_{\mathrm{Li}}^{2+}$-OH-${\mathrm{Er}}_{\mathrm{Nb}}^{2-}$ defect. By calculating the concentration of OH^−^-groups, we can select the required section of the gradient crystal cLiNbO_3_:Er^3+^(0.55 at%/cm) with the required conductivity value. In general, the analysis of changes in the vibrational spectra of the gradient crystal cLiNbO_3_:Er^3+^(0.55 at%/cm) will allow us to establish concentration thresholds within which the crystal structure is preserved. Numerical estimates of these boundaries will allow us to grow a crystal with the highest possible Er^3+^ concentration required for laser properties and minimal defects that can be redistributed within the crystal during intense heat generation during laser transitions.

## 4. Conclusions

Based on the analysis of the IR transmission spectra in the region of OH^−^-group stretching vibrations, it was established for the first time that the oxygen-octahedral MeO_6_ clusters in the structure of the LiNbO_3_:Er^3+^(3.1 wt%) single-crystal and the gradient crystal LiNbO_3_:Er^3+^(congruent composition by the main components, Er gradient of 0.55 at%/cm), as well as the oxygen-octahedral MeO_6_ clusters of the highly perfect compositionally homogeneous crystal of stoichiometric composition, have a shape close to the correct one, i.e., they are practically undistorted. The obtained results make it clear why the compositionally homogeneous LiNbO_3_:Er^3+^(3.1 wt%) single-crystal and the gradient crystal LiNbO_3_:Er^3+^(congruent composition by the main components, Er gradient of 0.55 at%/cm) have a low photorefraction effect: they have a low concentration of Nb_Li_ defects. The correctness of this conclusion is also confirmed by the absence of a second-order vibrational spectrum (IR transmission spectrum and Raman spectrum) of the compositionally homogeneous LiNbO_3_:Er^3+^(3.1 wt%) single-crystal in the frequency range of 1000–2000 cm^−1^, which is reliably manifested in the spectra of non-stoichiometric, compositionally homogeneous LiNbO_3_ crystals, nominally pure and doped. The gradient crystal LiNbO_3_:Er^3+^(congruent composition by the main components, Er gradient of 0.55 at%/cm) does not have a second-order IR transmission spectrum but has a second-order Raman spectrum. The presence of a second-order Raman spectrum can be associated with high microheterogeneity of the gradient crystal LiNbO_3_:Er^3+^(congruent composition by the main components, Er gradient of 0.55 at%/cm). By using the IR transmission spectra in the studied crystals, the volume concentration of OH^−^-groups was calculated using Klauer’s method. The concentration of OH^−^-groups in the gradient crystal LiNbO_3_:Er^3+^(congruent composition by the main components, Er gradient of 0.55 at%/cm) does not exceed 7.0 × 10^16^ cm^−3^. At the same time, the concentration of OH^−^-groups in the compositionally homogeneous crystal LiNbO_3_:Er^3+^(3.1 wt%) is 6.7 × 10^17^ cm^−3^, which is an order of magnitude greater than in the gradient crystal LiNbO_3_:Er^3+^(congruent composition by the main components, Er gradient of 0.55 at%/cm). This fact also indicates a lower conductivity of the gradient crystal LiNbO_3_:Er^3+^(congruent composition by the main components, Er gradient of 0.55 at%/cm) and, accordingly, a lower photorefraction effect, compared to the compositionally homogeneous crystal LiNbO_3_:Er^3+^(3.1 wt%).

## Figures and Tables

**Table 1:** Calculated concentration of OH^−^-groups for different areas of the gradient crystal cLiNbO_3_:Er^3+^(0.55 at%/cm).

Area	Concentration of the OH^−^-Groups, cm^−3^
1	2.62 × 10^16^
2	6.51 × 10^16^
3	4.21 × 10^16^
4	4.98 × 10^16^
5	3.23 × 10^16^
6	3.42 × 10^16^
7	8.33 × 10^15^
8	2.86 × 10^16^
9	1.90 × 10^16^

## Data Availability

The raw data required to reproduce these findings are available from the corresponding author, A.P., upon reasonable request.
